# Involvement of CsWRKY70 in salicylic acid-induced citrus fruit resistance against *Penicillium digitatum*

**DOI:** 10.1038/s41438-020-00377-y

**Published:** 2020-10-01

**Authors:** Bing Deng, Wenjun Wang, Changqing Ruan, Lili Deng, Shixiang Yao, Kaifang Zeng

**Affiliations:** 1 College of Food Science, Southwest University, 400715 Chongqing, China; 2 Research Center of Food Storage & Logistics, Southwest University, 400715 Chongqing, China

**Keywords:** Plant physiology, Biotic, Plant signalling, Effectors in plant pathology

## Abstract

*Penicillium digitatum* causes serious losses in postharvest citrus fruit. Exogenous salicylic acid (SA) can induce fruit resistance against various pathogens, but the mechanism remains unclear. Herein, a transcriptome-based approach was used to investigate the underlying mechanism of SA-induced citrus fruit resistance against *P. digitatum*. We found that *CsWRKY70* and genes related to methyl salicylate (MeSA) biosynthesis (salicylate carboxymethyltransferase, SAMT) were induced by exogenous SA. Moreover, significant MeSA accumulation was detected in the SA-treated citrus fruit. The potential involvement of CsWRKY70 in regulating *CsSAMT* expression in citrus fruit was studied. Subcellular localization, dual luciferase, and electrophoretic mobility shift assays and an analysis of transient expression in fruit peel revealed that the nucleus‐localized transcriptional activator CsWRKY70 can activate the *CsSAMT* promoter by recognizing the W-box element. Taken together, the findings from this study offer new insights into the transcriptional regulatory mechanism of exogenous SA-induced disease resistance in *Citrus sinensis* fruit.

## Introduction

Citrus fruit is threatened by a variety of pathogens, and the decay caused by *Penicillium digitatum* infections accounts for most of the economic losses during postharvest storage^1,2^. In practice, the extensive use of artificial fungicides, including thiabendazole, sodium *o*-phenyl phenate, and imazalil, has effectively inhibited the spread of citrus postharvest disease^3–5^. However, the emergence of resistant strains of pathogens and undesirable influences on the environment and human health caused by the wide use of these chemical fungicides have led to an increasing and urgent need for efficient and healthy alternatives to fungicide usage^6^.

Salicylic acid (SA) is one of the most important hormones in plants. Although the regulatory roles of SA in plant resistance to abiotic stress, growth, and development have been widely demonstrated, the most well known is its pivotal role in plant defense responses^7,8^. In flowering plants, SA is synthesized in either the cinnamic acid pathway or the chorismate pathway^9^. SA and its conjugates formed via methylation (methyl salicylate (MeSA)) and glycosylation (SA 2-*O*-*b*-d-glucoside) have been determined to be necessary for plant defense responses^10^. Apart from its positive role in host defense responses, applied SA influences the disease resistance of postharvest fruits^11–14^. The application of exogenous SA conferred resistance to many monocotyledonous and dicotyledonous plants to a variety of pathogenic microorganisms, such as viruses, bacteria, and fungi, and the underlying mechanisms were attributed to the deposition of callose plugs (reinforcements of plant cell walls), the synthesis of H_2_O_2_ (initiators of hypersensitive responses) and the accumulation of pathogenesis-related proteins (induce toxicity in microbes upon contact)^11–14^. For postharvest fruits, the application of exogenous SA may enhance the activity of disease resistance-related enzymes (β-1,3-glucanase, phenylalanine, and peroxidase), induce the expression of pathogenesis-related genes, and further enhance the disease resistance of postharvest fruits^6,12,15^. Although many studies have been conducted to determine the mechanism of SA-induced host resistance, few studies have focused on the underlying molecular mechanism of the transcriptional regulation in postharvest fruit.

The *WRKY* gene family is one of the largest transcription factor (TF) families in higher plants^16,17^. WRKY TFs (WRKYs) contain a DNA-binding domain that includes one or two highly conserved WRKYGQK motifs that follow a specific zinc-finger motif in the C terminus^16,17^. WRKYs participate in many biological processes, the best known of which is their pivotal role in plant defense responses^18^. According to a previous study, many WRKYs have been found to participate in SA-induced host resistance^19–25^. Exogenous SA can induce the expression of *Arabidopsis thaliana WRKY3*, *WRKY72*, *WRKY38/62*, and *WRKY46* and *Oryza sativa WRKY30*, *WRKY45*, and *WRKY23*, among others^19–25^. These SA-induced WRKYs may enhance plant disease resistance by activating the transcription of defense-related genes or repressing the transcription of development-relevant genes^26–28^. Nevertheless, the regulatory role of WRKYs in the postharvest disease resistance of citrus fruit remains unexplored.

In this study, the effect of SA at different concentrations on citrus fruit resistance against *P. digitatum* was investigated. Transcriptome analyses on SA-treated and control citrus fruit were carried out to better understand the underlying mechanism. Based on RNA-sequencing (RNA-seq) analysis, SA-responsive *CsWRKY70* was identified, and the possible association of CsWRKY70 with the direct activation of *CsSAMT* was investigated. This work provides new information on the mechanism of exogenous SA-induced host resistance in citrus fruits.

## Results

### Effect of exogenous SA on the disease resistance of citrus fruit against *P. digitatum*

**Fig. 1:**
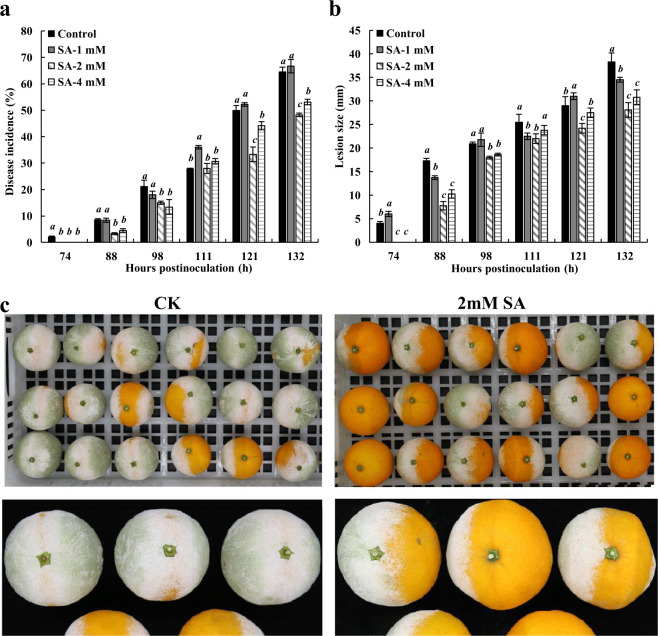
Effects of exgenous SA on the disease incidence and lesion size of citrus fruit caused by *P. digitatum*. **a** Changes of disease incidence. **b** Changes of lesion size. These two indicators were measured 74, 88, 98, 111, 121, and 132h post inoculation. Data represent the mean±s.e.m. of three biological replicates (*n*=3). **c** Appearance of the control and 2mM SA-treated citrus fruit at the late stage of disease

Decay caused by fungus accounts for the greatest loss of citrus fruit in postharvest storage^15,29^. In this study, we investigated the effect of different concentrations of SA on the disease incidence and lesion size of citrus fruit. As shown in Fig. 1a, the disease incidence of 2 mM SA-treated fruits was only 34%, while that of the control was 51% when measured 121 h post inoculation (h.p.i.). Compared with the control group, 2 and 4 mM SA treatment inhibited the enlargement of the lesion size, and 2 mM was the most effective SA concentration. In a previous study, the application of 2 mM SA obviously inhibited the development of green mold, which is consistent with our study^6^.

### Transcriptome analysis reveals the underlying mechanism of the SA-induced resistance of citrus fruit

**Fig. 2:**
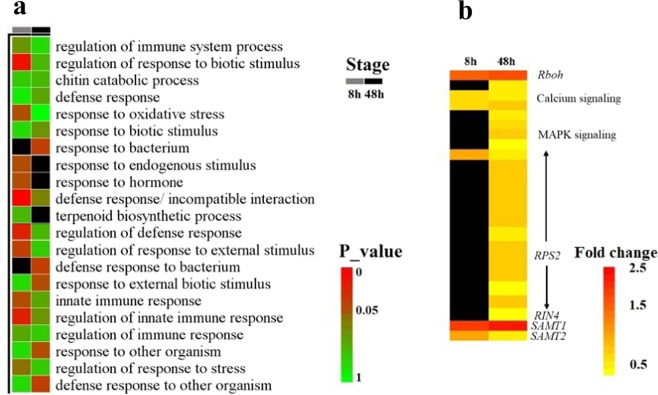
Analysis of DEGs related to disease resistance. **a** Heat map of Gene Ontology terms related to disease resistance as identified for upregulated genes. The red cubes indicate high enrichment factors for the corresponding gene functions, and the black cubes indicate that the corresponding gene functions are not enriched. **b** Heat map of the genes related to disease responses. Red represents a high fold change, and black represents genes that are not differentially expressed

To obtain a better understanding of the underlying mechanism of SA-induced citrus fruit resistance against *P. digitatum*, transcriptome analyses of citrus fruit 8 and 48 h.p.i. were performed. A Gene Ontology (GO) enrichment analysis showed that the differentially expressed genes (DEGs) induced by SA were enriched in many disease resistance-related gene functions (Fig. 2a). In addition, we analyzed the differential expression levels of genes correlated with immune responses (detailed information for the DEGs involved in immune responses is listed in Supplementary Table S2). As shown in Fig. 2b, several genes correlated with PAMP (pathogen-associated molecular pattern)-triggered immunity (PTI) were responsive to SA 8 h.p.i., including genes encoding the respiratory burst oxidase-like protein (which plays a pivotal role in the accumulation of reactive oxygen species) and cyclic nucleotide-gated ion channel 1 protein, which mediate the signal transduction of plant PTI. In addition to PTI-related DEGs, several genes encoding R proteins, which are critical for recognizing fungal effector proteins, were differentially expressed in the later stage (48 h.p.i.). Remarkably, two genes encoding salicylate carboxymethyltransferase (SAMT) were found to be differentially expressed in SA-treated citrus fruit (the Log_2_ fold change of these genes is listed in Supplementary Table S2). In higher plants, the methyl esterification of SA can be catalyzed by SAMT to produce MeSA^30,31^.

**Fig. 3:**
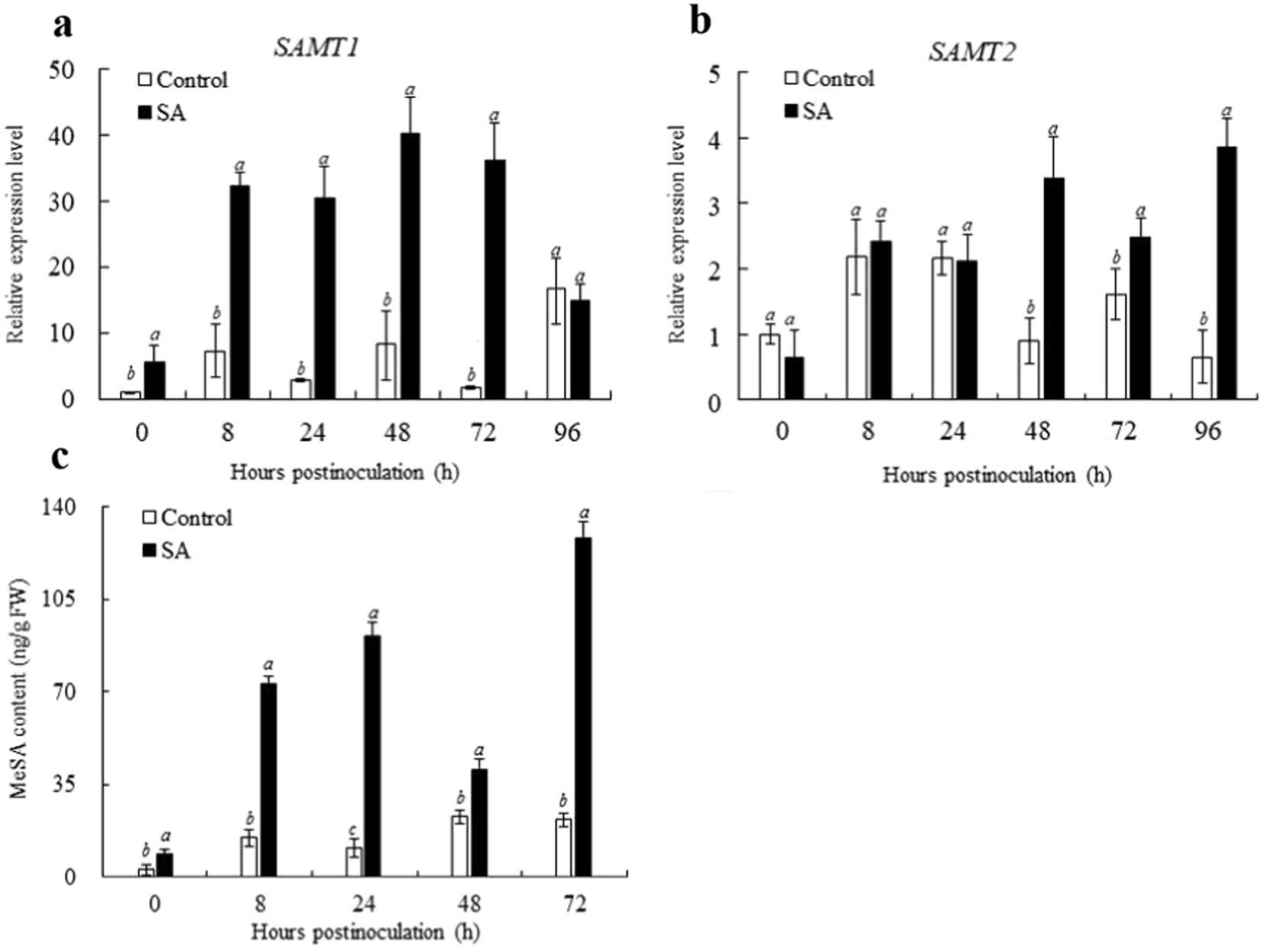
Effects of exogenous SA on the synthesis of MeSA upon pathogen inoculation. **a** The expression level of *CsSAMT1*. **b** The expression level of *CsSAMT2*. **c** Changes in the endogenous MeSA content. Citrus fruits were treated with 2 mM SA or water (control) following harvest, and then 10 μL of *P. digitatum* spores (5 × 10^4^ spores/mL in suspension) was inoculated, and the fruits (incubated at 25 °C in 90–95% relative humidity) were sampled 0, 8, 24, 48, 72, and 96 h.p.i. Differential expression levels were determined by qRT-PCR. *Citrus sinensis* actin (XM_006464503) was employed as the housekeeping gene, and the expression level of each *CsSAMT* gene was calculated as the ratio relative to the control sample at 0 h.p.i. (set as 1). Each value represents the mean ± s.e.m. of three replicates (*n* = 3)

MeSA acts as a phloem-mobile signal to establish systemic acquired resistance^32,33^. To investigate whether the accumulation of MeSA is influenced by exogenous SA, the changes in MeSA content in the citrus fruit during the storage period were measured. As shown in Fig. 3c, the endogenous MeSA content was notably (*P* < 0.05) enhanced by the SA treatment. In particular, at 8, 24, and 72 h.p.i., the MeSA content in the SA-treated fruits was over 2-, 4-, and 3.5-fold higher than that in the controls at 8, 24, and 72 h, respectively. The qRT-PCR analysis showed that the expression levels of *CsSAMTs* were largely consistent with the changes in MeSA. These results suggest that exogenous SA can induce the synthesis of MeSA in citrus fruit.

### Characterization of CsWRKY70

**Fig. 4:**
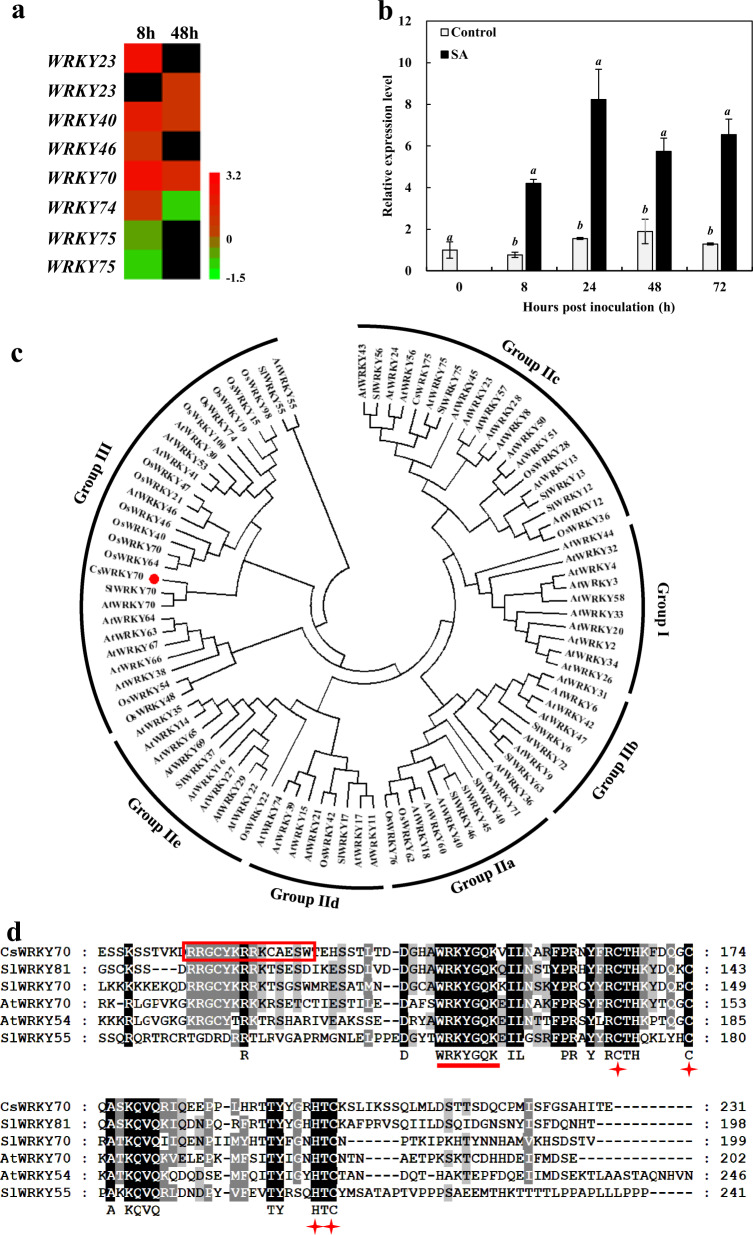
Characterization of CsWRKY70 in citrus fruit disease resistance against *P. digitatum* following exogenous SA treatment. **a** Differentially expressed transcription factor genes. **b** Transcript level of *CsWRKY70* in citrus fruit with disease resistance against *P. digitatum* after exogenous SA treatment. **c** Phylogenetic tree of CsWRKY70, *O. sativa*, *S. lycopersicum*, and *A. thaliana* WRKYs. CsWRKY70 (red circle), along with *A. thaliana* AtWRKY70, *S. lycopersicum* SlWRKY70, *O. sativa* OsWRKY70, and *O. sativa* OsWRKY64 clusters in group III. **d** Relationships between CsWRKY70 and other plant WRKY family proteins. Identical amino acid sequences in different WRKYs are represented by black shading. The WRKY motif is underlined, the nuclear localization signal is marked in a red box, and the zinc-finger structure is indicated by asterisks

WRKYs perform critical roles in host disease resistance to biotic stresses, and many WRKYs have been identified as important regulators of SA-induced host resistance^18^. Given that exogenous SA induces MeSA accumulation, we were interested in the underlying transcriptional mechanism. We conducted transcriptome analysis of citrus fruit in response to different stimulants (SA and other phytohormones), and the results showed that *CsWRKY70* was specifically responsive to exogenous SA (unpublished data). As shown in Fig. 4a, *CsWRKY70* was significantly upregulated at both time points (the Log_2_ fold change at 8 and 48 h.p.i. was 2.3 and 3.4, respectively, as shown in Supplementary Table S2). To confirm the specificity of CsWRKY70 in SA-induced citrus fruit resistance against *P. digitatum*, the expression of *CsWRKY70* was measured by reverse transcription-quntitative PCR (RT-qPCR (Fig. 4b). In accordance with its trend in the transcriptome data, the *CsWRKY70* expression level upon SA treatment increased after pathogen infection, which implies that CsWRKY70 participated actively in the SA-induced immune responses of the citrus fruit.

According to the RNA-seq and *C. sinensis* genome databases (http://citrus.hzau.edu.cn/ orange), the full-length *CsWRKY70* was cloned. Through a homology search, we found that its sequence shared the highest identity with *Solanum lycopersicum* SlWRKY70 (4-c). *CsWRKY70* contains a complete open-reading frame (ORF) of 984 bp, which encodes a protein of 328 amino acids, and the predicted molecular weight and isoelectric point of CsWRKY70 were 36.7 and 6.32 kDa, respectively. The amino acid sequence analysis revealed that CsWRKY70 presented one highly conserved zinc-finger containing the WRKY DNA-binding domain (60 amino acids, extending from 141 to 200) in the C-terminal region, and the zinc-finger pattern was C-X7-C-X23-H-X1-C (Fig. 4d). According to the common classification methods based on *A. thaliana*, WRKYs in plants can be classified into three groups (I, II, and III) based on their number of WRKY domains and the zinc-finger-like motif features^18^. The phylogenetic tree constructed with CsWRKY70 and model plant WRKYs showed that CsWRKY70, SlWRKY70, and AtWRKY70 had the same pattern of conserved group III WRKY proteins.

### Analysis of the subcellular localization and transcriptional activity of CsWRKY70

**Fig. 5:**
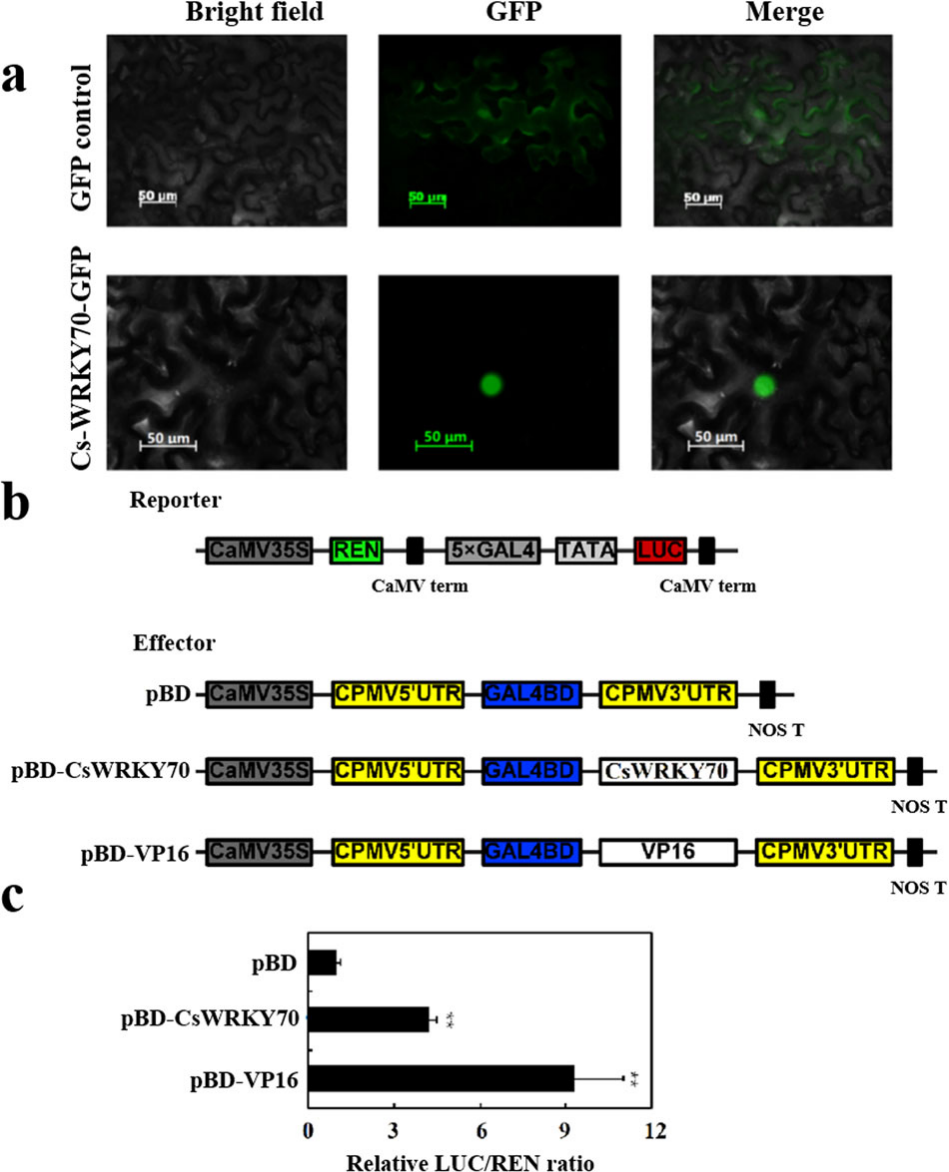
Subcellular localization and transcriptional activity analyses of CsWRKY70. **a** Subcellular localization of CsWRKY70 in tobacco leaves. *Agrobacterium tumefaciens* carrying CsWRKY70-GFP or GFP vector (positive control) were infiltrated into tobacco leaves. The fluorescent signal was observed 60 h post infiltration. The fluorescence of CsWRKY70-GFP protein was observed only in the nucleus, while the fluorescence of the positive control was distributed throughout the whole cell. Bar = 50 μM. **b** Schematics of the reporter and effector constructs used in the DLR experiment. **c** Transcriptional activation level of CsWRKY70. The transactivation capacity of CsWRKY70 was assessed as the ratio of LUC to REN. The ratio of LUC/REN in the empty pBD vector was used as a standard and set at 1. **Significant difference between the sample and the control, *P* < 0.01

TFs are produced in the cytoplasm, and the transport of TFs from the cytoplasm to the nucleus mediated by the recognition nuclear localization signals (NLSs) by transporter proteins is a necessary step for posttranslational regulation^34,35^. A predicted NLS at the N terminus (119–132) implies that CsWRKY70 is translocated into the nucleus to regulate gene transcription (Fig. 4d). To investigate the subcellular location of CsWRKY70, the coding sequence of *CsWRKY70* was fused with the gene encoding green fluorescent protein (GFP) in a pEAQ plasmid, and the construct was transiently expressed in tobacco leaves. As shown in Fig. 5, the GFP fluorescence of the CsWRKY70-GFP fusion protein was present only in the nucleus, while in the positive control, the GFP signal was observed throughout the cytosol and nucleus. The results indicated that CsWRKY70 was localized to the nucleus.

Depending on the transcriptional regulation ability, TFs can be categorized as transcriptional activators or transcriptional repressors^36^. A dual-luciferase reporter system was used to examine the transcriptional activity of CsWRKY70 in tobacco leaves. Firefly luciferase (LUC) fused with five GAL4 DNA-binding elements plus a TATA box and Renilla luciferase (REN) driven by the 35 S promoter constituted the reporter, and *CsWRKY70* fused with the GAL4 DNA-binding domain in the pBD vector was used as the effector (Fig. 5b). As shown in Fig. 5c, the LUC/REN ratios of pBD-VP16 (transcriptional activator control) and pBD-CsWRKY70 were all significantly higher than those of pBD. These results indicated that CsWRKY70 was a transcriptional activator localized in the nucleus.

### CsWRKY70 specifically binds to the *CsSAMT* promoter and activates the expression of *CsSAMT* genes

**Fig. 6:**
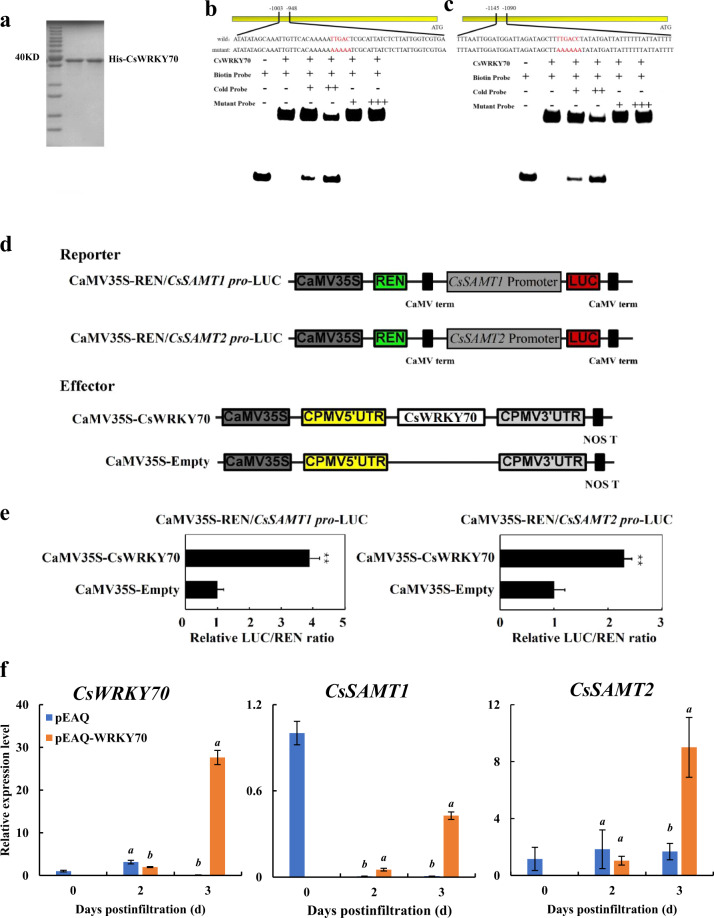
CsWRKY70 specifically binds to the *CsSAMT* promoter and activates the expression of *CsSAMTs*. **a** SDS–PAGE was conducted to detect the purification of the recombinant CsWRKY70 protein. **b**, **c** EMSA. Biotin-labeled probe or mutant probe was incubated with His-CsWRKY70 protein, and the mixtures were separated on a 6% native polyacrylamide gel. The symbols + and + + indicate increasing amounts of unlabeled probe used for competition with or increasing the amounts of mutant probe used to test the binding specificity. **d** Schematics of the reporter and effector constructs used in the DLR experiment. The *CsSAMT* promoter was inserted into a PGREENII 0800-LUC vector (*Kpn*I and *Nco*I) as a reporter, and the full-length *CsWRKY70* was fused to a PEAQ vector (*Age*I and *Xho*I) as an effector. Each pair of effector and reporter plasmids was transformed into an *A. tumefaciens* strain and then transiently expressed in tobacco leaves (*Nicotiana benthamiana*). **e** CsWRKY70 transactivates the *CsSAMT* promoters. Activation was indicated by the ratio of LUC to REN. The ratio of LUC/REN of the empty vector plus promoter was used for calibration and set to 1. Each value represents the mean ± s.e.m. (*n* = 5). **Statistically significant differences at *P* < 0.01. **f** Effect of transient *CsWRKY70* expression on endogenous MeSA biosynthesis genes (*CsSAMTs*). Error bars indicate standard errors (SEs) from three replicates

The WRKY domain can bind to target genes by recruiting the W-box in the promoter region (*cis*-acting element)^18^. In a previous study, many W-box sequences were specifically bound to WRKY TFs, and the combination was essential for the expression of target genes^37–39^. A motif scan of the *CsSAMT* genes revealed W-boxes in the promoters of both *CsSAMT* genes, which implied that the expression of *CsSAMT* might be regulated by WRKY TFs. Remarkably, qRT-PCR revealed that the *CsWRKY70* and *CsSAMT* genes had similar expression patterns during the storage period. Based on the above-mentioned results, we inferred that *CsSAMTs* might be the target genes of CsWRKY70. To confirm this hypothesis, an electrophoretic mobility shift assay (EMSA) was conducted to verify the binding specificity of the recombinant CsWRKY70 to the *CsSAMT* promoter. The recombinant His-CsWRKY70 protein was efficiently expressed in the *Escherichia coli* expression system and purified with Ni-NTA chromatography (Fig. 6a). As shown in Fig. 6b, c, the band shifts were observed when the CsWRKY70 protein was incubated with each biotin-labeled probe (containing W-boxes), which indicated that CsWRKY70 can directly bind to these sequences. The CsWRKY70 can bind to the biotin-labeled probes and the corresponding unlabeled probe, with which it competes, but not to the corresponding unlabeled mutant probe (containing the mutated W-box). These results suggested that CsWRKY70 specifically was bound to the *CsSAMT* promoter.

CsWRKY70 is a transcriptional activator, and it can specifically bind to the *CsSAMT* promoter. These results imply that CsWRKY70 may positively regulate the expression of *CsSAMTs*. To verify this supposition, the ability of CsWRKY70 to activate the transcription of the *CsSAMT* genes was detected through transient dual-luciferase assays. As shown in Fig. 6d, the *CsSAMT1* and *CsSAMT2* promoters were inserted into a pGreenII 0800-LUC vector to serve as reporters. *CsWRKY70* was inserted into a pEAQ vector to serve as an effector, and pEAQ was used as a control. As shown in Fig. 6e, the transient expression of *CsWRKY70* significantly enhanced the LUC/REN ratio of the reporters containing *CsSAMTs* relative to that of the corresponding empty control.

Due to the long juvenile period of citrus, it is very difficult to verify the function of CsWRKY70 in vivo by using gene-silencing technology or stable overexpression lines. To better illustrate the regulatory effects of CsWRKY70 on the *CsSAMT* genes, transient overexpression of *CsWRKY70* in citrus fruit peels was conducted. As shown in Fig. 6f, the transient overexpression of *CsWRKY70* resulted in the expression of *CsSAMT1* and *CsSAMT2* 3 days post infiltration. These results suggested that CsWRKY70 can activate the expression of *CsSAMTs*.

## Discussion

*Penicillium digitatum* is one of the most destructive postharvest pathogens of citrus fruit^2^. Some chemical treatments, such as 2,4-d, thiabendazole and imazalil, have been widely used to inhibit the spread of the disease^5^. However, considering the potential hazards to human health and the environment of the above-mentioned methods, it is necessary to find an environmentally friendly and effective method^6^. The induction of host resistance is a promising alternative to avoid the negative side effects of chemical treatments^6^. As a pivotal signaling molecule, SA plays pivotal roles in plant immune responses against a variety of biotic stresses^8^. Moreover, it has been validated that exogenous SA has positive effects on inducing the host resistance of many plants and postharvest fruits^6,11,15,40^. However, the applicability of SA for inducing citrus fruit disease resistance and the underlying molecular mechanism are still unclear.

### Exogenous SA can induce natural resistance of citrus fruit

A previous study confirmed that the application of 2 mM SA significantly decreased the decay of Satsuma mandarin (*Citrus unshiu*) fruit during postharvest storage^6^. In the present study, the effect of different concentrations of SA on the disease resistance of *C. sinensis* fruit against *P. digitatum* was examined. The results suggested that a 2 mM SA treatment is a potent method to inhibit the occurrence of green mold. Transcriptome analyses showed that SA treatment can enhance host immune responses against *P. digitatum* by inducing the expression of various disease resistance genes. Remarkably, two *CsSAMT* genes, which are essential for the formation of MeSA, were identified as differentially expressed in SA-treated citrus fruit. More importantly, the endogenous MeSA level in the SA-treated samples was significantly higher than that of the control fruit after pathogen infection. These results implied that exogenous SA can induce the accumulation of MeSA in citrus fruit. In a previous study, 0.2 mM SA was applied to transformed *Atropa belladonna* roots induced the accumulation of MeSA and methyl-*o*-methoxy-benzoate^41^. Further research revealed that AbSAMT1 mRNA began to be expressed 12 h post exposure, and steady expression continued over 144 h^41,42^. The accumulation of MeSA might contribute to SA-induced disease resistance.

### CsWRKY70 participates in the transcriptional regulation of SA-induced disease resistance

WRKY TFs are best known for their transcriptional regulation of plant disease resistance^18^. In previous studies, the involvement of five WRKY III subfamily *AtWRKYs* in *A. thaliana* immune responses was verified, and all of these TFs (*AtWRKY38*, *AtWRKY46*, *AtWRKY53*, *AtWRKY62*, and *AtWRKY70*) were induced by either *Pseudomonas syringae* or exogenous SA^20,43^. Further research showed that AtWRKY70 plays a synergistic role in the SA-signaling-mediated immune responses of *A. thaliana*, which suggests the possible involvement of CsWRKY70 in the SA-induced disease resistance of citrus fruit^43^.

In a previous study, the transcription of *AtWRKY70* was activated by exogenous SA and inhibited by exogenous JA^44^. Further studies confirmed that the constitutive overexpression of *AtWRKY70* induced the constitutive expression of SA-induced pathogenesis-related genes (*PR1*) and, in contrast, suppressed *AtWRKY70*-activated JA-responsive genes (*PDF1.2*)^44,45^. These results suggest that AtWRKY70 acts as an activator of SA-induced genes and a repressor of JA-responsive genes^44,45^. In addition, the expression of *PtrWRKY89* (the homolog of AtWRKY70) was induced by exogenous SA in *Populus trichocarpa*^46^. Moreover, the overexpression of *PtrWRKY89* induced the expression of the SA-signaling pathway-responsive *PRs*^46^. Notably, the overexpression of *AtWRKY70* or its homologous genes did not cause changes to the SA levels or to SA biosynthesis genes^45–47^. These results suggested that WRKY70 is actively involved in SA-mediated disease resistance and acts downstream of the SA-dependent signaling pathway.

In this study, we found that CsWRKY70 is a group III WRKY TF. Further research showed that the nucleus-localized transcriptional activator CsWRKY70 can specifically bind to two *CsSAMT* promoters to activate the expression of the *CsSAMT* genes. This result provides a new basis to elucidate the underlying mechanism of exogenous SA-induced disease resistance in citrus fruit.

## Materials and methods

### Plant material and postharvest treatment

Mature citrus fruit (*C. sinensis* L. Osbeck cv. Jincheng 447#) was harvested from an orchard in the Beibei district of Chongqing, China. Fruits of uniform size were selected and randomly divided into two groups for further experiments. For SA treatments, citrus fruits were submerged in 1, 2, or 4 mmol/L SA aqueous solutions containing 0.1% Tween-80 for 30 min. Citrus fruits dipped in distilled water containing 0.1% Tween-80 for 30 min constituted the control. Each treatment was performed with three biological replicates with ~50 fruits in each replicate. Both the control and SA-treated citrus fruits were then rinsed with distilled water and subsequently air-dried at room temperature. After 24 h, all the fruits were wounded at two opposite points near the equator (3-mm wide × 3-mm deep), and then 10 μL of a *P. digitatum* suspension (5 × 10^4^ spores/mL) was inoculated at each point. All fruits were then packed in plastic bags separately and stored at 25 °C and 90–95% relative humidity. The disease incidence and lesion size were recorded every 12 h according to a previous study^48^. Immediately after spore suspension was inoculated and after 8, 24, 48, 72, and 96 h of storage, the pericarp (8 mm around the inoculation site) of three replicates with five fruits were collected for further analysis.

### RNA isolation and RNA-seq

Approximately 1 g of sample from each sampling point was used to extract total RNA following the protocol described by Bugos et al.^49^. The extraction buffer was modified from the original and consisted of 100 mmol/L Tris, 0.2 mmol/L NaCl, 15 mmol/L EDTA, 0.5% sodium dodecyl sulfate (SDS), and 1% β-mercaptoethanol.

Control samples were selected for RNA-seq 0, 8 and 48 h.p.i., and SA-treated samples were selected for RNA-seq 8 and 48 h.p.i. The RNA-seq and bioinformatics analyses were conducted by Novogene (Beijing, China). The method use for the library construction, RNA-sequencing and RNA-seq data analysis were consistent with comparable methods described in a previous study^48^.

### cDNA synthesis, quantitative real‐time PCR (RT-qPCR) and gene cloning

Approximately 1.0 μg of RNA was used to produce cDNA with an iScript gDNA Clear cDNA synthesis kit (1725034, Bio-Rad, USA). RT-qPCR was performed on a CFX96 real-time PCR system (Bio-Rad, USA) with iTaq Universal SYBR Green Supermix (1725121, Bio-Rad, USA). The PCRs and thermal cycling conditions were consistent with methods described in a previous study^48^. *Citrus sinensis* actin (XM_006464503) was used as a reference gene, and the relative expression quantity of each gene was calculated by the 2^−ΔΔCt^ method^50^.

Based on the *C. sinensis* genome database (http://citrus.hzau.edu.cn/orange/), the coding sequence containing the intact ORF of *CsWRKY70* was verified by PCR and cloned into a T cloning vector. ClustalW and GeneDoc software was used for multiple alignment assays. A phylogenetic tree consisting of WRKY proteins was constructed using the MEGA6.0 software.

### Genomic DNA extraction and promoter cloning

Genomic DNA was extracted from young citrus leaves using a plant genomic DNA kit (DP305, TIANGEN). With genomic DNA as a template, the promoter sequences containing *cis*-elements of *CsSAMTs* were amplified by PCR. The online JASPAR database (http://jaspar.genereg.net/) was used to analyze the *cis*-element motifs in the promoters.

### Quantification of endogenous MeSA content

The extraction and quantification of endogenous MeSA content were based on the method described in a previous study^51^. For endogenous hormone extraction, ~1.0 g of citrus peels (three replicates) were ground into powder in liquid nitrogen and homogenized in 1 mL of extraction buffer (2-propanol:H_2_O:concentrated HCl = 2:1:0.002) for 1 h at 4 °C^51^. Endogenous MeSA was quantified by high-performance liquid chromatography-mass spectrometry using an Agilent 6460 Triple Quad system with an Agilent‐XDB C18 column (2.1 mm × 150 mm). Samples were injected onto the reversed‐phase column using a binary solvent system composed of distilled water with 0.1% (vol/vol) formic acid (A) and with methanol with 0.1% (vol/vol) formic acid (B) as a mobile phase. The oven (holding high-performance liquid chromatography column) temperature and solvent flow rate were 40 °C and 0.3 mL/min, respectively. The mass spectrometer was operated in the positive mode using the following source settings: capillary voltage, 130 V (electrospray ionization (ESI)); desolvation temperature, 600 °C; and source temperature, 150 °C. Argon was used as the collision gas at a flow rate of 0.15 mL/min. MRM mode was used to monitor the precursor‐to‐product ion transition with a 50 MS dwell time, and the precursor ions were fragmented with a collision energy of 23 V, and products in the range 120–153*m*/*z* were scanned. The ultra-performance liquid chromatography–ESI–tandem mass spectrometry system control, data acquisition, and data analyses were performed with Agilent MassHunter Workstation software. Seven concentration points, 100, 200, 300, 500, 600, 800, and 1000 ng/mL, were used to establish calibration curves (shown in Supplementary Fig. S1).

### Subcellular localization

Using the recombinant T plasmid as a template, the intact coding sequence without the stop codon for *CsWRKY70* was amplified by using specific primers and then fused to a pEAQ-GFP vector (*Age*I). The recombinant plasmid (pEAQ-CsWRKY70-GFP) was introduced into an *A. tumefaciens* strain (EHA105) and then transiently expressed in 5-week-old tobacco (*N. benthamiana*) leaves according to the method of a previous study^36^. In addition, *A. tumefaciens* harboring a pEAQ‐GFP plasmid was transiently expressed in tobacco leaves as the control. The GFP signal was visualized with an Axioskop 2 Plus fluorescence microscope (Zeiss, Jena, Germany) 48–60 h post infiltration.

### Transient transcriptional activation assay

By using a dual-luciferase transient expression system, the transcriptional activity of CsWRKY70 and the regulatory role of CsWRKY70 on target promoters were investigated. For the assay of CsWRKY70 transcription activity, the full-length sequence without the stop codon for *CsWRKY70* was recombined to a pBD vector (*Stu*I) as an effector, and the modified PGREENII 0800-LUC vector was adopted as the reporter, which contained firefly luciferase and was driven by the CaMV35S promoter with a 5-GAL-binding element and *Renilla* luciferase driven by CaMV35S. To study the regulatory role of CsWRKY70 on the *CsSAMT* promoters, full-length *CsWRKY70* was fused to a pEAQ vector (*Age*I and *Xho*I) as an effector, and the *CsSAMT1* and *CsSAMT2* promoters were fused separately to the PGREENII 0800-LUC vector (*Kpn*I and *Nco*I) as the reporter.

All the effector plasmids were transferred into *A. tumefaciens* (EHA105), and all the reporter plasmids were transferred into *A. tumefaciens* (EHA105 (pSOUP)). The cultures were adjusted to an OD600 of 0.6 with an infiltration buffer (150 mM acetosyringone; 10 mM MES; and 10 mM MgCl_2_, pH 5.6). After a 3-h incubation at 25 °C, the effecter and corresponding reporter in *A. tumefaciens* were mixed at a proportion of 9:1, and the mixtures infiltrated into tobacco (*N. benthamiana*) leaves. The plants were cultivated in a glasshouse for 60–72 h, and the LUC and REN activities were assayed with a dual-luciferase assay kit (Promega).

### Electrophoretic mobility shift assay

The full-length *CsWRKY70* was fused to a pCold I vector (*Kpn*I and *Pst*I) to construct the His-CsWRKY70 expression vector, and the recombinant plasmid was transformed into *E. coli* strain BM Rosetta (DE3). His-CsWRKY70 was induced by 0.1 mM isopropyl β-d-1-thiogalactopyranoside at 15 °C for 24 h, and the recombinant protein was purified by the elution of gradient imidazole-containing buffers. SDS-polyacrylamide gel electrophoresis was used to evaluate the efficiency of the purification process. Probes containing a W-box in the promoters of *CsSAMT* were end labeled with a biotin 3′-end DNA labeling kit (Thermo Scientific, 89818). EMSA was performed using a LightShift chemiluminescent EMSA kit (Thermo Scientific, 20148).

### Analysis of the transient expression of the *CsSAMT* genes in citrus fruit peel

By using the pEAQ-CsWRKY70 construct, a transient expression experiment was conducted in citrus fruit peel (*C. sinensis*). The culture of *A. tumefaciens* (EHA105) carrying pEAQ-CsWRKY70 was adjusted to an OD600 of 0.5 with an infiltration buffer (150 mM acetosyringone; 10 mM MES; and 10 mM MgCl_2_, pH 5.6). After 3 h of incubation at 25 °C, the culture was infiltrated into citrus peels. The citrus fruits were stored at 25 °C for 72 h, and the peels were collected every 24 h to detect the expression level of *CsWRKY70* and *CsSAMT* genes.

## Supplementary information

Supplementary Information
