# Supplementary figures and images for: Involvement of CsWRKY70 in salicylic acid-induced citrus fruit resistance against Penicillium digitatum
Source: Hortic Res. 2020 Oct 1;7:157. doi: 10.1038/s41438-020-00377-y (PMC7527965; doi:10.1038/s41438-020-00377-y)

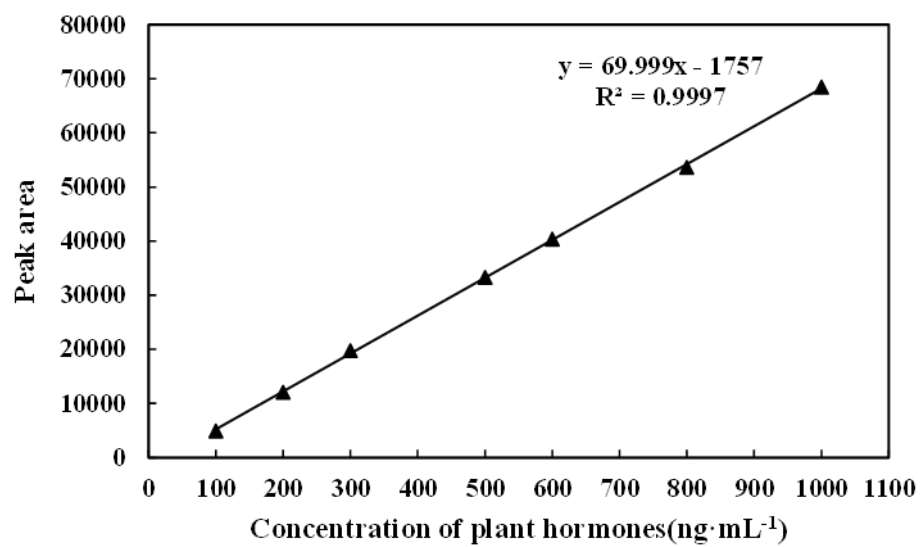

Figure-S1 Calibration curve used for the calculation of MeSA content

Supplement: Supplementary file 1 — Supplementary Information [file 41438_2020_377_MOESM1_ESM.pdf]
